# Beta-catenin activation and immunotherapy resistance in hepatocellular carcinoma: mechanisms and biomarkers

**DOI:** 10.20517/2394-5079.2020.124

**Published:** 2021-01-07

**Authors:** Sandi A. Kwee, Maarit Tiirikainen

**Affiliations:** 1 Cancer Biology Program (SAK) and Population Sciences in the Pacific Program (MT), University of Hawaii Cancer Center, University of Hawaii, Honolulu, Hawaii 96813, USA.; 2 Hamamatsu/Queen’s PET Imaging Center, The Queen’s Medical Center, Honolulu, Hawaii 96813, USA.

**Keywords:** Hepatocellular carcinoma, immunotherapy, immune checkpoint, biomarkers, positron emission tomography, beta-catenin

## Abstract

Mutations involving CTNNB1, the gene encoding beta-catenin, and other molecular alterations that affect the Wnt/beta-catenin signaling pathway are exceptionally common in hepatocellular carcinoma. Several of these alterations have also been associated with scarcity of immune cells in the tumor microenvironment and poor clinical response to immune checkpoint inhibitor therapy. In light of these associations, tumor biomarkers of beta-catenin status could have the potential to serve as clinical predictors of immunotherapy outcome. This editorial review article summarizes recent pre-clinical and clinical research pertaining to associations between beta-catenin activation and diminished anti-tumor immunity. Potential non-invasive biomarkers that may provide a window into this oncogenic mechanism of immune evasion are also presented and discussed.

## BACKGROUND

Hepatocellular carcinoma (HCC) is frequently diagnosed at an advanced clinical stage or precluded from surgery by poor underlying liver function^[1,2]^. Patients with clinically-advanced HCC have poor survival, with estimated one-year survival rates of 21% for those with regionally advanced disease and 6% for those with metastatic disease^[1]^. After a decade of meager advances in systemic therapy, the treatment of unresectable advanced-stage HCC has taken a leap forward with the advent of immune checkpoint inhibitor (ICI) antibodies. In the United States, the first ICI agent to gain regulatory approval for HCC was the anti-programmed death 1 (anti-PD1) monoclonal antibody nivolumab. Accelerated approval for this ICI as a second-line systemic agent for HCC came after a 20% objective response rate was reported in the dose expansion phase of its phase I/II trial (CheckMate-040) that involved patients who were refractory to, or intolerant of, sorafenib^[3]^. Pembrolizumab, another anti-PD1 antibody, also received accelerated approval as a second-line therapeutic after similar response rates were reported in a non-randomized open-label phase II clinical trial (KEYNOTE-224)^[4]^. Most recently, a regimen combining bevacizumab with atezolizumab, an anti-programmed death ligand 1 (PD-L1) antibody, received clinical approval as a first-line systemic therapy for unresectable HCC, based on its phase III trial (IMbrave150) reporting an objective response rate of 36%, along with better overall survival compared with sorafenib, the prior standard of care^[5]^.

Notwithstanding the hope that immunotherapy brings to patients with advanced HCC, all trials to date have shown substantial heterogeneity in the degree to which tumors respond to these immune-oncology agents. In the recently completed double-blind placebo-controlled randomized phase III trial of pembrolizumab (KEYNOTE-240), only 2% of treated patients experienced a complete response (CR), while 16% and 32% experienced partial response (PR) and disease progression (PD), respectively^[6]^. These results mirror closely what was reported in the phase II trial of pembrolizumab (CR in 1%, PR in 16%, and PD in 33%)^[4]^. Similarly, in the CheckMate-040 trial of nivolumab, 1%, 18%, and 32% of patients experienced CR, PR, and PD, respectively^[3]^. This degree of heterogeneity in tumor response has since been recapitulated in observational cohorts of HCC patients receiving IC therapy^[7,8]^. The observational data also suggest that heterogeneity in tumor response translates into heterogeneity in survival outcome^[7]^. Clinical response has also been heterogeneous in the first-line treatment setting, with the IMbrave150 trial reporting 5.5% CR, 21.8% PR, and 19.8% PD in Child-Pugh class A patients not previously treated with sorafenib^[5]^. Durations of clinical response are also highly variable, ranging 6-24 (median 17) months in the CheckMate-040 trial and 1.5-23.6 (median 13.8) months in the KEYNOTE-240 trial^[3,9]^. Since immune-related adverse events can be serious, a predictive biomarker that can adequately explain this heterogeneity across patients would be of great benefit in the clinical setting for optimizing patient selection.

Unfortunately, no clinical trial to date has identified a reliable tissue or serum biomarker to predict immunotherapy response in HCC. Although tumor PD-L1 expression has been associated with anti-PD1/PD-L1 ICI response in multiple cancers (including non-small cell lung, bladder, cervical, and triple-negative breast cancer), this immunohistochemical marker has not yet proven to be a good predictor of response for HCC^[3,4]^. Furthermore, while high microsatellite instability (MSI-H) and tumor mutation burden (TMB) have been shown to be predictive of clinical immunotherapy response in multiple other cancers, they have not proven very predictive of ICI response in HCC^[8]^. It is therefore time to start looking beyond these immediate immunotherapy biomarkers. Examining what is known about the underlying mechanisms of tumor immune evasion in HCC and other cancers may help facilitate a broader search for immunotherapy biomarkers that are useful for HCC.

## ONCOGENIC MECHANISMS OF TUMOR IMMUNE EVASION LEADING TO POOR IMMUNOTHERAPY RESPONSE

Multiple lines of mechanistic and clinical research have identified potential associations between tumor immunotherapy resistance and well-known oncogenic pathways and mechanisms^[10-18]^. Of these, the Wnt/β-catenin signaling pathway was among the earliest to be associated with tumor immune suppression^[13,19]^. In a genetically-engineered mouse model of melanoma that exhibits stabilized β-catenin expression, it was first discovered that aberrant activation of this pathway could lead to the disappearance of immune cells from the tumor microenvironment (TME) and the development of tumor resistance to anti-PD-L1/anti-CTLA-4 monoclonal antibody therapy^[13,20]^. β-catenin expression in this model was also associated with decreases in the expression of immune-oncology relevant chemokines such as CCL4 that are important for recruiting dendritic cells and consequently T-cells into the TME. The mechanism by which β-catenin reduces CCL4 expression was tied to the induction of ATF3, a transcriptional repressor, and its binding to the CCL4 promoter^[13,20,21]^. This immune evasion mechanism has since been recapitulated in a similarly engineered model of β-catenin activated HCC, where it was observed that aberrant β-catenin activation led to tumor resistance to anti-PD1 therapy and that reinstating the expression of CCL5, a chemokine that was downregulated in the β-catenin driven tumors, could restore tumor immune surveillance^[22]^.

TME infiltration by antigen presenting dendritic cells and T-cells, leading to a so-called inflamed TME, is now recognized as a critical factor that allows anti-PD1 and anti-PD-L1 antibodies to exert their therapeutic effects^[21,22]^. Across numerous cancer types, analysis of TCGA data has revealed a significant difference in the percentage of Wnt/β-catenin activating mutations between immune inflamed and non-inflamed tumors, with the largest differences observed in HCC and esophageal cancer^[12]^. Histologically, the TME of β-catenin activated HCC also displays a paucity of immune cells^[23]^. Hence, one may speculate that it may be possible to infer the status of anti-tumor immunity in HCC by analyzing tumor biomarkers that capture the status of β-catenin activation.

## WNT/β-CATENIN SIGNALING

**Figure 1.**
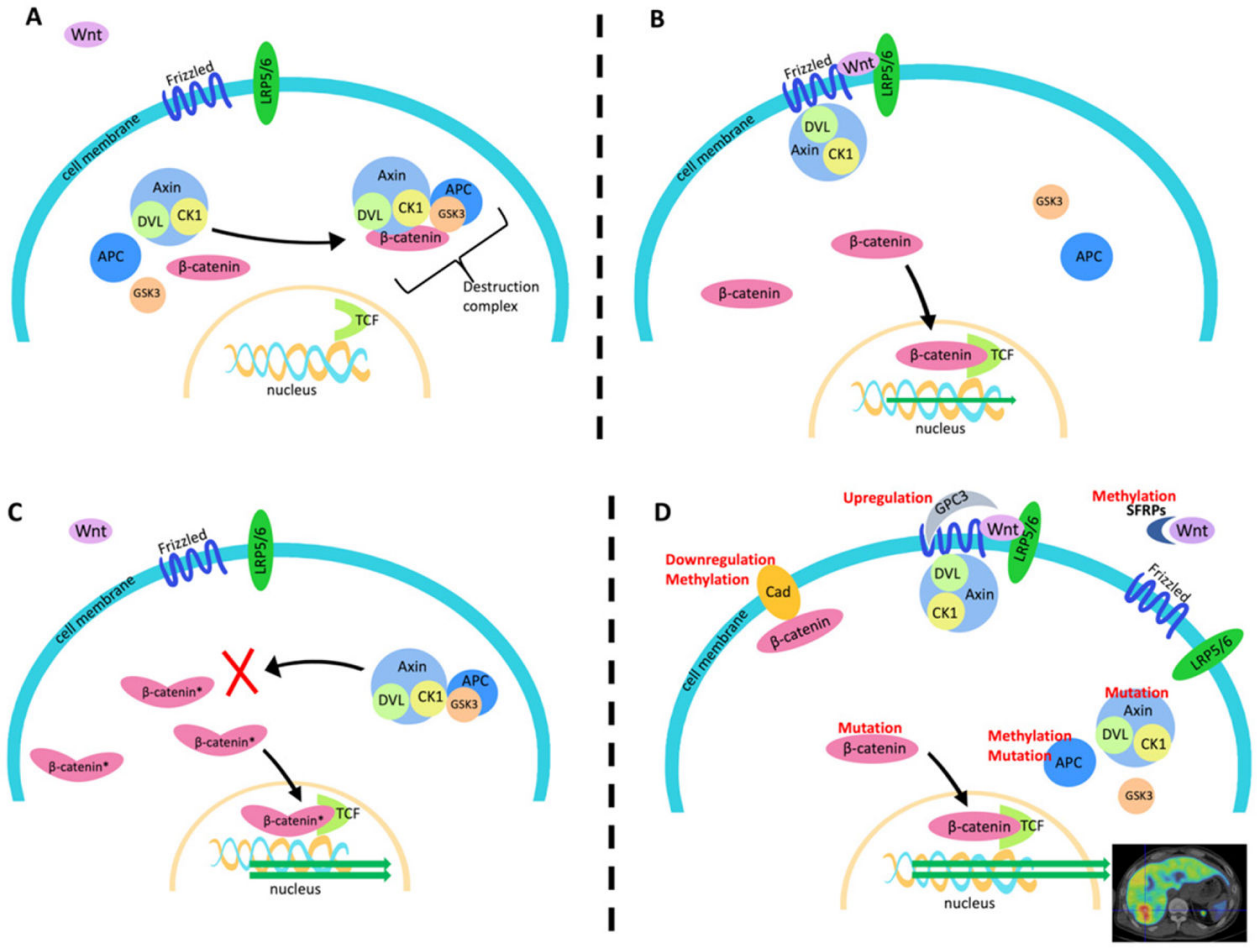
Mechanism of Wnt/β-catenin signaling and potential molecular alterations that may affect β-catenin signaling. A: In the absence of Wnt binding at the cell membrane, β-catenin is kept in check by a large destruction complex comprised of Axin, DVL, CK1, GSK3, and APC; B: Wnt binding to Frizzled and LRP5/6 sequesters Axin and its associated molecules increases the abundance of unphosphorylated β-catenin to enhance classical Wnt/β-catenin signaling; C: as the most frequent causes of aberrant β-catenin signaling in HCC, *CTNNB1* exon 3 mutations protect β-catenin from GSK3-mediated phosphorylation, leading to an increase in the amount of stable β-catenin that can enter the nucleus; D: in addition to mutations, other molecular alterations (indicated in red) can lead to aberrant Wnt/β-catenin activity to consequently promote a myriad of changes in tumor phenotype. Lipoprotein receptor related proteins 5 and 6 (LRP5/6), dishevelled protein (DVL), adenomatous polyposis coli (APC), casein kinase 1 (CK1), glycogen synthase kinase 3 (GSK3), T cell factor (TCF), glypican-3 (GPC-3), soluble frizzled related protein (SFRP), and E-cadherin (Cad)

The Wnt/β-catenin signaling pathway is highly conserved across species and constitutively involved in embryonic development, cell migration, and tissue homeostasis^[24]^. This pathway has been shown to play multiple roles in tumorigenesis and tumor immune-evasion^[12,25-29]^. In the canonical Wnt/β-catenin pathway, the nuclear transcription co-activator protein β-catenin undergoes continual degradation in the cytoplasm by a destruction complex comprised of the scaffolding protein Axin (*AXIN1*) along with casein kinase 1 (*CK1*), glycogen synthase kinase 3 (*GSK3*), and the adenomatous polyposis coli (*APC*) gene product [Figure 1A]^[30]^. Wnt binding at the cell membrane by Frizzled protein and low-density lipoprotein receptor-related proteins 5 and 6 (*LRP5/6*) promotes recruitment of this Axin complex to the cell membrane via dishevelled (DVL) proteins. Membrane bound, this β-catenin destruction complex is no longer able to mediate the phosphorylation, ubiquitination, and subsequent proteasomal degradation of β-catenin^[31]^. As a consequence, the stable form of β-catenin accumulates in the nucleus where it can interact with DNA-bound T cell factor/lymphoid enhancer factor (TCF/LEF) proteins to promote Wnt target gene expression [Figure 1B]^[32]^.

Aberrant activation of this pathway in cancer can be caused by a number of different molecular mechanisms, including gene mutations, epigenetic alterations, and abnormal regulation of other pathways. Mutations involving catenin-beta-1 (*CTNNB1*), the gene that encodes for β-catenin, are among the most common causes in HCC, as they are found in approximately one-third of HCC tumors^[33-36]^.

Mutations involving other Wnt/β-catenin pathway-related genes can also lead to aberrant β-catenin signaling^[12,33,35,36]^. For example, the *AXIN1* gene is mutationally inactivated in 5%-19% of HCC tumors, making it the second most frequently mutated gene leading to aberrant Wnt/β-catenin activation in HCC^[35,37]^. Molecular alterations external to the classical Wnt/β-catenin pathway can also contribute to enhanced β-catenin signaling [Figure 1D], including overexpression of glypican-3 (GPC3)^[38,39]^, downregulation of E-cadherin^[40]^, and extracellular Wnt inhibition by secreted Frizzled-related proteins (FRZBs or SFRPs)^[11,12,41]^

## β-CATENIN ACTIVATING MUTATIONS IN HCC

Several gene mutations consistently overrepresented in HCC are associated with the β-catenin activated state^[12,33,35,36,42]^. In particular, mutations involving *CTNNB1* occur in an estimated 25%-40% of HCC tumors^[12,16,23,36]^ The specific *CTNNB1* mutations that have been associated with an immune-barren tumor landscape are almost exclusive to areas within exon 3. These areas correspond to coding regions for phosphorylation sites near the N-terminus of β-catenin that are involved in mediating its breakdown^[34]^. Mutations involving these regions allow β-catenin, a transcription co-activator, to escape destruction and accumulate in the nucleus, where binding to T-cell factor/lymphoid enhancing factor allows it to aberrantly promote transcriptional programs that include those that may influence tumor immune phenotype^[43-45]^.

Consistent with this apparent mechanism of β-catenin mediated immune cell exclusion, clinical associations between markers of Wnt/β-catenin activation and various molecular signs of immunotherapy resistance have been revealed through numerous and exhaustive molecular profiling and genotyping studies of human tumor specimens^[10,12,16,17,46]^.

Mutated *CTNNB1* and other mutated genes known to cause Wnt/β-catenin activation have been associated with poor clinical response to treatment with ICI antibodies and other targeted agents in HCC^[12,16,33,34]^. In one study that included 27 ICI-treated HCC patients who underwent pre-treatment tumor mutation profiling, 10/10 of HCC patients with tumors bearing *CTNNB1* or *AXIN1* mutations experienced progressive disease compared to 5/17 patients with tumors that did not have these β-catenin activating mutations (100% *vs*. 29%, *P* < 0.001)^[16]^. The gene *AXIN1* codes for a protein involved in β-catenin destruction^[12,30,36,37,42,47]^. The specific *AXIN1* mutations involved cause loss-of-function, and they are usually mutually exclusive to *CTNNB1* mutations^[47,48]^. In addition to experiencing higher rates of progressive disease, ICI-treated HCC patients with *CTNNB1* or *AXIN1* mutated tumors also experience significantly shorter progression free survival (median 2.0 months *vs*. 7.4 months, HR = 9.2, 95%CI: 2.9-28.8, *P* < 0.0001)^[16]^. These clinical associations imply that the mutation statuses of these genes may have some value as predictive or prognostic biomarkers for HCC immunotherapy.

Several other gene mutations that can drive Wnt/β-catenin signaling are relatively uncommon in HCC^[12,28,33,35]^. For example, mutations that result in the loss or inactivation of the adenomatous polyposis coli (APC) gene product are a frequent cause of aberrant β-catenin activation in colon and rectal cancer. However, while it is estimated that over 80% of colorectal cancers harbor APC mutations, they are relatively uncommon in HCC with an estimated frequency of 5%-7%^[16,49]^. With the recent clinical approval of two cell-free DNA (cfDNA) sequencing panels for mutation profiling with relatively broad (pan-cancer) coverage, it may now be practical to detect these mutations along with the more prevalent ones through non-invasive liquid biopsy. In addition to profiling a large set of cancer-associated genes, these panels also generate blood-based estimates of TMB (bTMB) and MSI-H status, making it conceivable that an integrative genomic profile of relevance to the HCC immunotherapy space may be discovered. Cohort studies as well as carefully designed prospective biomarker trials will be necessary to learn, test, and validate such mutational profiles as predictors of ICI response. Since HCC is a cancer for which tumor tissue is difficult to obtain clinically, these pan-cancer liquid biopsy panels may eventually prove to be a boon to the development of more precise targeted therapy for this disease. However, regarding immunotherapy, mutation status alone may not be able to fully explain the estimated 60%-80% of HCC patients who fail to exhibit an objective response to ICI therapy.

## DOES β-CATENIN ACTIVATED HCC HAVE A METABOLIC PHENOTYPE?

Other molecular mechanisms, including epigenetic alterations^[48]^, as well as alterations involving extracellular pathways^[38-40,48]^, have also been associated with aberrant Wnt/β-catenin signaling in HCC. There are also data suggesting that upregulated lipid metabolism in HCC is associated with β-catenin activation^[28,29,50]^. This association raises the possibility of inferring tumor β-catenin activation status based on phenotypic assessments of tumor metabolism. Such assessments are clinically feasible and can be performed non-invasively using several molecular imaging methods including positron emission tomography (PET). As a proof of concept with regard to phospholipid metabolism, a recent series of experiments performed in an adenomatous polyposis coli (APC) knock-out mouse model of β-catenin activated HCC suggests that tumor uptake of fluorocholine (FCH), a fluorine-18 labeled PET radiopharmaceutical analog of choline used for *in-vivo* tracing of tissue phosphatidylcholine synthesis^[51,52]^, is strongly influenced by β-catenin activation^[28]^. This novel animal model of *de-novo* HCC can develop well, moderately, and poorly differentiated tumors that are capable of recapitulating the salient histological and molecular features of β-catenin-driven human HCC^[53]^. Experimentally, increased tumor FCH uptake in this system was shown to correlate with increased β-catenin protein expression as well as metabolomic and transcriptional fingerprints of canonical Wnt/β-catenin activity. Conversely, control animal models with non-β-catenin-activated tumors were found to demonstrate low tumor FCH uptake^[28]^. Providing a simple mechanistic explanation for how this pathway promotes increased tumor FCH uptake and choline phospholipid metabolism, it was confirmed that β-catenin activation was able to drive choline membrane transporter expression. Following a translational route, the team that conducted these experiments also obtained and sequenced tumor DNA from 13 patients that had underwent clinical FCH PET/CT and found mutated *CTNNB1* in 6/7 patients with FCH-positive tumors and wild-type *CTNNB1* in 6/6 patients with FCH-negative tumors^[28]^.

**Figure 2.**
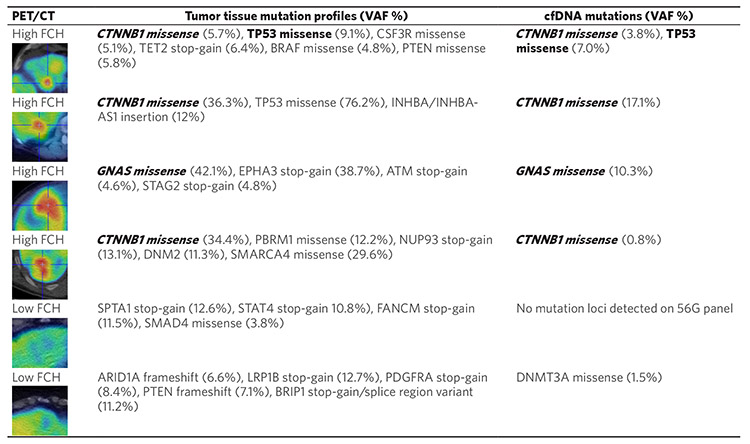
Comparison of mutation profiling results obtained from pre-treatment cfDNA and post-resection tumor DNA in six HCC patients imaged by 18F-FCH PET/CT preoperatively. Mutations that show correspondence between tumor DNA and cfDNA are shown in bold. Mutations associated with aberrant Wnt/β-catenin pathway activation are italicized. On the liver PET/CT images shown in the first column, the tumors displaying high FCH uptake appear red on the rainbow color scale applied to these images. Note: A *DNMT3A* mutation found in the cfDNA but not the tumor DNA of one patient (bottom row) is likely due to clonal hematopoiesis of indeterminate potential (CHIP). CHIP-associated mutations arise from hematopoietic cells and are age-related. They are often encountered incidentally through cfDNA sequencing^[55,56]^. DNMT3A is the most frequently mutated gene associated with CHIP^[57,58]^. VAF: variant allele frequency

In an attempt to corroborate their results, we examined the tumor genomic profiling results from six HCC patients who underwent preoperative liver FCH PET/CT imaging prior to liver resection. These patients were among the participants of a recently completed diagnostic clinical trial of FCH PET/CT in liver cancer^[54]^. To explore whether β-catenin activating mutations found in tumor DNA could also be detected in cfDNA, we analyzed the pre-treatment plasma samples collected from these patients by performing targeted mutation profiling of cfDNA using an oncology-specific next generation sequencing panel (56G Oncology Panel, Swift Biosciences). The results of this liquid biopsy mutation analysis along with the PET imaging and clinical tumor DNA profiling results are shown in Figure 2.

As a summary of these preliminary findings, mutations associated with Wnt/β-catenin activation were detected in the tumor DNA of four patients, with three being *CTNNB1* mutations and one being a mutation involving the guanine nucleotide binding protein-alpha stimulating sub-unit (*GNAS*) gene. These same genes were also found mutated in the corresponding cfDNA of these patients. Interestingly, mutations involving *GNAS* have been reported to be a cause of upregulated Wnt/β-catenin activity and lipid metabolism in several digestive tract cancers but not yet in HCC^[59,60]^. Notably, the tumors of all four of these patients showed high uptake of FCH on PET/CT (image insets on Figure 2). Conversely, no Wnt/β-catenin activating mutations were identified in the tumor DNA or cfDNA of the remaining 2 patients. The tumors of both these patients showed low FCH uptake on PET/CT. Interestingly, one of these FCH non-avid tumors harbored a *SMAD4* gene mutation associated with Wnt/β-catenin pathway downregulation^[61]^. While these limited data do not allow us to make any statistical conclusions, they do appear to agree with the results obtained by the other investigators and, furthermore, provide a demonstration of the potential capability of liquid biopsy to detect Wnt/β-catenin activating tumor mutations in HCC.

**Figure 3.**
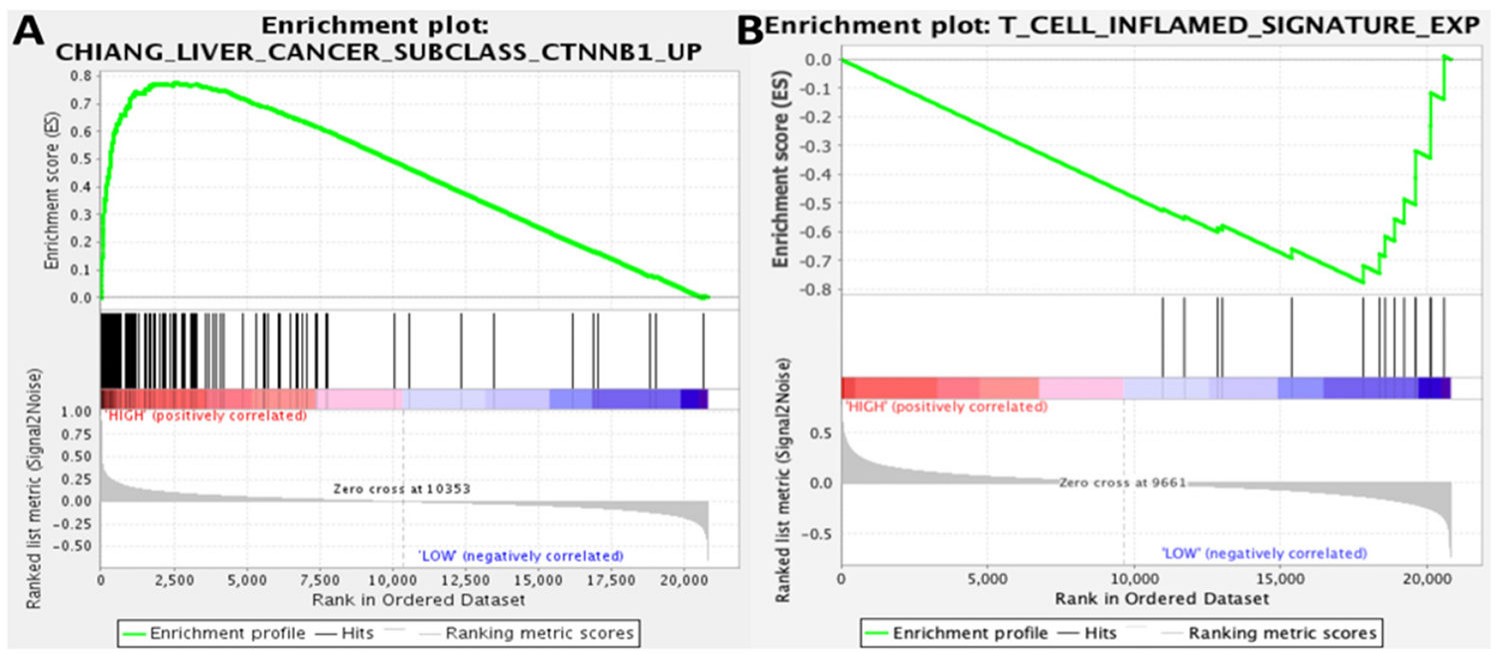
Gene set enrichment analysis associates tumor FCH uptake with immunotherapy-relevant expression profiles. Gene set enrichment plots are based on 41 tumor samples (31 FCH-avid, 10 FCH non-avid): (A) tumors showing high FCH metabolism were significantly enriched for genes from a *CTNNB1* activation signature (FDR 0.062); and (B) a signature of T-cell inflammation that can predict immunotherapy response in several different tumor types was enriched by tumors that showed low FCH metabolism (FDR 0.116)

**Figure 4.**
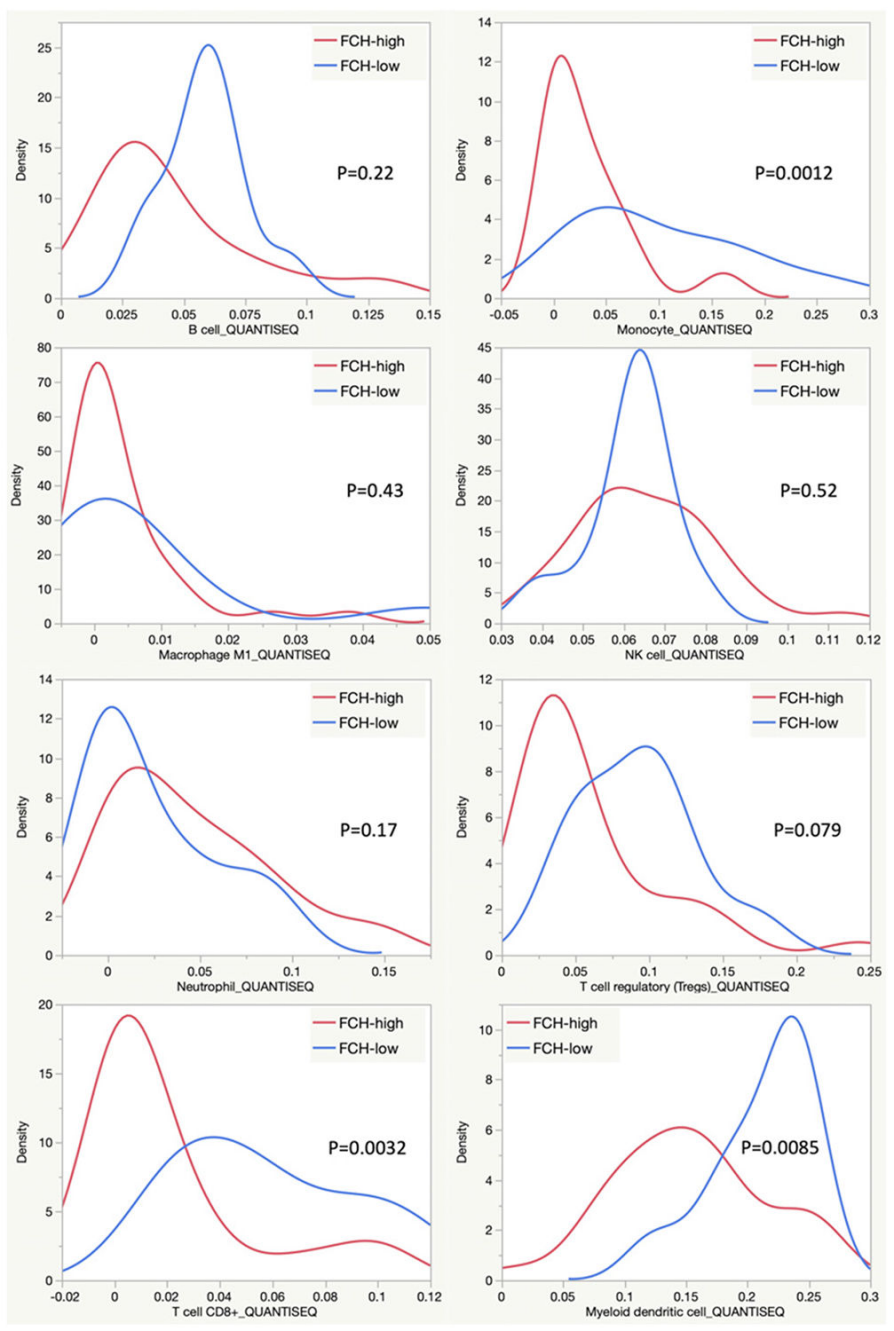
QuantiSeq TIMER estimation of intratumoral immune cell abundance. Density plots compare estimates of immune cell infiltration between tumors demonstrating high FCH uptake (*n* = 31) and low FCH uptake (*n* = 10). The names of the immune cell lineages are identified below the X-axis of these plots

In further corroboration of these findings, we revisited a whole-genome expression dataset generated from 41 HCC tumors imaged preoperatively by FCH PET/CT^[54]^. Using gene set enrichment analysis, we found that sets of genes associated with β-catenin activation were significantly enriched by FCH-avid HCC tumors (FDR 0.062) [Figure 3A]. Conversely, we found tumors displaying low FCH uptake were enriched by a T-cell inflammation signature that has been shown to be strongly predictive of clinical response to ICI therapy in several different cancers (FDR 0.116) [Figure 3B]^[15]^. Furthermore, our previous published analysis of this radiogenomic dataset^[54]^ reported that tumors displaying high FCH uptake disproportionately expressed gene signatures corresponding to distinct molecular classes of HCC, including the S3 class described by Hoshida *et al*.^[62]^; the G5 and G6 classes described by Boyault *et al*.^[63]^; and the “*CTNNB1*-activated” class described by Chiang *et al*.^[64]^. All of these classes have more recently been associated with newly described immunotherapy-relevant HCC sub-types characterized by an immunosuppressed TME or poor ICI response as well as evidence of abnormal β-catenin activity^[10,46,65]^. One recently described immune-suppressed type of HCC shows significant overlap with the Hoshida S3 class and is notable for its association with a lack of TME infiltration by immune cells as well as a high likelihood of being *CTNNB1* mutated^[46]^. A recent integrative analysis of DNA methylation and gene expression revealed another sub-type of HCC that showed enrichment for *CTNNB1* mutations and signatures of Wnt activation but lacked signs of immune-activation^[65]^. This tumor sub-type showed significant overlap with both the Hoshida S3 class and the Chiang *CTNNB1*-activated class that were characterized by high tumor FCH uptake in our studies. The Boyault G5 and G6 classes, among the earliest to be associated with *CTNNB1* mutations^[63]^, have also been recently implicated with poorly immunogenic sub-types^[10,65]^. This high degree of transcriptomic overlap forms an intriguing link between tumor FCH avidity and poor anti-tumor immunity. Furthermore, a TIMER (Tumor Immune Estimation Resource^[66]^, accessed via timer.cistrome.org) based analysis of the tumor expression profiles revealed significantly higher estimated densities of monocytic, CD8+, and dendritic cells among the tumors that displayed low FCH uptake [Figure 4], suggesting that poor FCH avidity was associated with immune cell infiltration. While collectively these results support associations between Wnt/β-catenin activation, lipid metabolism, and tumor immune-evasion in HCC, it remains to be tested in clinical trials whether molecular imaging biomarkers such as those derived from FCH PET/CT can serve as reliable predictors of immunotherapy response for HCC.

## PERSPECTIVES ON THE CLINICAL FUTURE OF IMMUNOTHERAPY BIOMARKERS IN HCC

In 2019, the KEYNOTE-240 phase III trial was reported to have failed in achieving its pre-determined statistical endpoints for survival^[6]^. However, durations of clinical response in the trial ranged from 1.5 months to 23.6 months, and the risk of death overall was reduced by 22% (HR = 0.781, 95%CI: 0.611-0.998, *P* = 0.0238). The implication of these results is that some patients will benefit substantially from these agents, but the benefits will be thinly spread across too many patients in the absence of a robust predictive biomarker that can be used to refine patient selection. Because anti-PD1 agents can lead to prolonged disease control in those who do respond, a predictive biomarker of treatment resistance/response could have substantial value in both the clinical and research domains. For clinical trials, a reliable predictive biomarker may substantially reduce the study sample size required and increase the statistical power for an a-priori treatment effect size^[67]^. Because immune-related adverse events to ICI therapy are non-trivial, bringing such a biomarker to the clinical practice space would help guide patients with vulnerable tumors to appropriate therapy while protecting those who are unlikely to respond from the hazards of futile treatment and its side effects. From a healthcare economics standpoint, a predictive biomarker would help to enhance the value proposition of ICI treatment by reducing costs associated with wasted treatments and unhalted disease progression.

However, robust biomarkers, detectors, predictors, and other classifiers that are singular in nature are rare in the field of cancer. There are multiple biological and statistical reasons for why an integrative biomarker would perform better than a single or narrowly targeted set of biomarkers^[35,65,68-70]^. An understanding of how different non-convergent molecular pathways and phenotypes can shape tumor immunity in HCC may support multimarker integration as an approach to predicting immunotherapeutic response^[10,35,46,65,70]^. There is also a growing number of statistical learning and machine learning based approaches to building, integrating, and evaluating multi-biomarker classifiers^[68,71,72]^, although the optimal method for assigning significance to any incremental gains in biomarker classification performance has been a topic of debate^[73,74]^. The tools for integrative biomarker design and analysis have also become research tools to elucidate the biologic origin and functional significance of different biomarkers with the potential of shedding more light on their clinical and biological importance.

The contemporary clinical approach to the diagnosis of HCC has evolved into something rather unique among solid tumors. Diagnostic algorithms for HCC, such as those based on the National Comprehensive Cancer Networks (NCCN) guidelines^[75]^, allow for the diagnosis of HCC to be predicated on the results of radiographic testing in appropriately selected patients. In those patients with cirrhosis or chronic liver disease, satisfaction of radiographic criteria based on contrast-enhanced CT or MRI (LIRADS-5) confers a positive predictive value of > 98% for the diagnosis of HCC, effectively alleviating the need for liver biopsies to secure a tissue diagnosis^[75]^. Unfortunately, this non-histopathologic approach to diagnosis can impede clinical and research efforts to advance precision medicine for HCC, as it makes tumor tissue unavailable for genetic testing or molecular profiling. Non-invasive diagnostic tools such as liquid biopsy and molecular imaging have the potential to address this lack of molecular information. Clinical development of such non-invasive methods for HCC for molecular-subclassification, risk-stratification, and treatment selection share the promise of preserving and extending the non-invasive clinical diagnosis and management approach pioneered in HCC.

## SUMMARY AND CONCLUSION

Presently, there is a critical yet unmet need for biomarkers to predict immunotherapeutic response in HCC, since objective clinical responses to the existing approved ICI agents occur in only a fraction of patients. There is now a large body of evidence that associates Wnt/β-catenin activation with tumor immune evasion and immunotherapeutic resistance. Recent work has also shown that it is feasible to assess the Wnt/β-catenin activation status of malignant tumors non-invasively through liquid biopsy and possibly molecular imaging. Further efforts along these lines to develop non-invasive assays of tumor Wnt/β-catenin activation may have the potential to be fruitful in producing much-needed biomarkers for predicting immunotherapeutic response in HCC. However, as with therapeutic agents, these biomarker tools will require rigorous and thorough clinical testing along a well-planned series of clinical trial phases that begins with the assessment of biomarker classification performance and ends with the measurement of clinical impact.

## References

[1] Altekruse SF, McGlynn KA, Reichman ME. Hepatocellular carcinoma incidence, mortality, and survival trends in the United States from 1975 to 2005. J Clin Oncol 2009;27:1485–91. 19224838 10.1200/JCO.2008.20.7753 PMC2668555

[2] Yarchoan M, Agarwal P, Villanueva A, Recent developments and therapeutic strategies against hepatocellular carcinoma. Cancer Res 2019;79:4326–30. 31481419 10.1158/0008-5472.CAN-19-0803 PMC8330805

[3] El-khoueiry AB, Sangro B, Yau T, Nivolumab in patients with advanced hepatocellular carcinoma (CheckMate 040): an open-label, non-comparative, phase 1/2 dose escalation and expansion trial. Lancet 2017;389:2492–502. 28434648 10.1016/S0140-6736(17)31046-2 PMC7539326

[4] Zhu AX, Finn RS, Edeline J, Pembrolizumab in patients with advanced hepatocellular carcinoma previously treated with sorafenib (KEYNOTE-224): a non-randomised, open-label phase 2 trial. Lancet Oncol 2018;19:940–52. 29875066 10.1016/S1470-2045(18)30351-6

[5] Finn RS, Qin S, Ikeda M, ; IMbrave150 Investigators. Atezolizumab plus bevacizumab in unresectable hepatocellular carcinoma. N Engl J Med 2020;382:1894–905. 32402160 10.1056/NEJMoa1915745

[6] Finn RS, Ryoo B, Merle P, ; for the KEYNOTE-240 Investigators. Results of KEYNOTE-240: phase 3 study of pembrolizumab (Pembro) vs best supportive care (BSC) for second line therapy in advanced hepatocellular carcinoma (HCC). JCO 2019;37:4004.

[7] Lee PC, Chao Y, Chen MH, Predictors of response and survival in immune checkpoint inhibitor-treated unresectable hepatocellular carcinoma. Cancers (Basel) 2020;12:182. 10.3390/cancers12010182 PMC7017111 31940757

[8] Ang C, Klempner SJ, Ali SM, Prevalence of established and emerging biomarkers of immune checkpoint inhibitor response in advanced hepatocellular carcinoma. Oncotarget 2019;10:4018–25. 31258846 10.18632/oncotarget.26998 PMC6592287

[9] Finn RS, Ryoo BY, Merle P, ; KEYNOTE-240 investigators. Pembrolizumab as second-line therapy in patients with advanced hepatocellular carcinoma in KEYNOTE-240: a randomized, double-blind, phase III trial. J Clin Oncol 2020;38:193–202. 31790344 10.1200/JCO.19.01307

[10] Shimada S, Mogushi K, Akiyama Y, Comprehensive molecular and immunological characterization of hepatocellular carcinoma. EBioMedicine 2019;40:457–70. 30598371 10.1016/j.ebiom.2018.12.058 PMC6412165

[11] Pinyol R, Sia D, Llovet JM. Immune exclusion-Wnt/CTNNB1 class predicts resistance to immunotherapies in HCC. Clin Cancer Res 2019;25:2021–3. 30617138 10.1158/1078-0432.CCR-18-3778 PMC6445700

[12] Luke JJ, Bao R, Sweis RF, Spranger S, Gajewski TF. WNT/β-catenin pathway activation correlates with immune exclusion across human cancers. Clin Cancer Res 2019;25:3074–83. 30635339 10.1158/1078-0432.CCR-18-1942 PMC6522301

[13] Spranger S, Bao R, Gajewski TF. Melanoma-intrinsic β-catenin signalling prevents anti-tumour immunity. Nature 2015;523:231–5. 25970248 10.1038/nature14404

[14] Taube JM, Young GD, McMiller TL, Differential expression of immune-regulatory genes associated with PD-L1 display in melanoma: implications for PD-1 pathway blockade. Clin Cancer Res 2015;21:3969–76. 25944800 10.1158/1078-0432.CCR-15-0244 PMC4558237

[15] Ayers M, Lunceford J, Nebozhyn M, IFN-γ-related mRNA profile predicts clinical response to PD-1 blockade. J Clin Invest 2017;127:2930–40. 28650338 10.1172/JCI91190 PMC5531419

[16] Harding JJ, Nandakumar S, Armenia J, Prospective genotyping of hepatocellular carcinoma: clinical implications of next-generation sequencing for matching patients to targeted and immune therapies. Clin Cancer Res 2019;25:2116–26. 30373752 10.1158/1078-0432.CCR-18-2293 PMC6689131

[17] Spranger S, Gajewski TF. Impact of oncogenic pathways on evasion of antitumour immune responses. Nat Rev Cancer 2018;18:139–47. 29326431 10.1038/nrc.2017.117 PMC6685071

[18] Ou Q, Yu Y, Li A, Association of survival and genomic mutation signature with immunotherapy in patients with hepatocellular carcinoma. Ann Transl Med 2020;8:230. 32309377 10.21037/atm.2020.01.32 PMC7154492

[19] Yaguchi T, Goto Y, Kido K, Immune suppression and resistance mediated by constitutive activation of Wnt/β-catenin signaling in human melanoma cells. J Immunol 2012;189:2110–7. 22815287 10.4049/jimmunol.1102282

[20] Spranger S, Dai D, Horton B, Gajewski TF. Tumor-residing Batf3 dendritic cells are required for effector T Cell trafficking and adoptive T cell therapy. Cancer Cell 2017;31:711–23.e4. 28486109 10.1016/j.ccell.2017.04.003 PMC5650691

[21] Spranger S, Gajewski TF. A new paradigm for tumor immune escape: β-catenin-driven immune exclusion. J Immunother Cancer 2015;3:43. 26380088 10.1186/s40425-015-0089-6 PMC4570721

[22] Ruiz de Galarreta M, Bresnahan E, Molina-Sánchez P, β-catenin activation promotes immune escape and resistance to anti-PD-1 therapy in hepatocellular carcinoma. Cancer Discov 2019;9:1124–41. 31186238 10.1158/2159-8290.CD-19-0074 PMC6677618

[23] Calderaro J, Couchy G, Imbeaud S, Histological subtypes of hepatocellular carcinoma are related to gene mutations and molecular tumour classification. J Hepatol 2017;67:727–38. 28532995 10.1016/j.jhep.2017.05.014

[24] Clevers H Wnt/beta-catenin signaling in development and disease. Cell 2006;127:469–80. 17081971 10.1016/j.cell.2006.10.018

[25] Audard V, Grimber G, Elie C, Cholestasis is a marker for hepatocellular carcinomas displaying beta-catenin mutations. J Pathol 2007;212:345–52. 17487939 10.1002/path.2169

[26] Nishida N, Nishimura T, Nagasaka T, Ikai I, Goel A, Boland CR. Extensive methylation is associated with beta-catenin mutations in hepatocellular carcinoma: evidence for two distinct pathways of human hepatocarcinogenesis. Cancer Res 2007;67:4586–94. 17510384 10.1158/0008-5472.CAN-06-3464

[27] Kim G, Kurnit KC, Djordjevic B, Nuclear β-catenin localization and mutation of the CTNNB1 gene: a context-dependent association. Mod Pathol 2018;31:1553–9. 29795437 10.1038/s41379-018-0080-0 PMC6168348

[28] Gougelet A, Sartor C, Senni N, Hepatocellular carcinomas with mutational activation of beta-catenin require choline and can be detected by positron emission tomography. Gastroenterology 2019;157:807–22. 31194980 10.1053/j.gastro.2019.05.069

[29] Senni N, Savall M, Cabrerizo Granados D, β-catenin-activated hepatocellular carcinomas are addicted to fatty acids. Gut 2019;68:322–34. 29650531 10.1136/gutjnl-2017-315448

[30] Hart MJ, de los Santos R, Albert IN, Rubinfeld B, Polakis P. Downregulation of β-catenin by human Axin and its association with the APC tumor suppressor, β-catenin and GSK3β. Current Biology 1998;8:573–81. 9601641 10.1016/s0960-9822(98)70226-x

[31] He X, Semenov M, Tamai K, Zeng X. LDL receptor-related proteins 5 and 6 in Wnt/beta-catenin signaling: arrows point the way. Development 2004;131:1663–77. 15084453 10.1242/dev.01117

[32] van de Wetering M, Cavallo R, Dooijes D, Armadillo coactivates transcription driven by the product of the drosophila segment polarity gene dTCF. Cell 1997;88:789–99. 9118222 10.1016/s0092-8674(00)81925-x

[33] Zucman-Rossi J, Villanueva A, Nault JC, Llovet JM. Genetic landscape and biomarkers of hepatocellular carcinoma. Gastroenterology 2015;149:1226–39.e4. 26099527 10.1053/j.gastro.2015.05.061

[34] Gao C, Wang Y, Broaddus R, Sun L, Xue F, Zhang W. Exon 3 mutations of *CTNNB1* drive tumorigenesis: a review. Oncotarget 2018;9:5492–508. 29435196 10.18632/oncotarget.23695 PMC5797067

[35] Li W, Wang H, Ma Z, Multi-omics analysis of microenvironment characteristics and immune escape mechanisms of hepatocellular carcinoma. Front Oncol 2019;9:1019. 31681571 10.3389/fonc.2019.01019 PMC6803502

[36] Schulze K, Imbeaud S, Letouzé E, Exome sequencing of hepatocellular carcinomas identifies new mutational signatures and potential therapeutic targets. Nat Genet 2015;47:505–11. 25822088 10.1038/ng.3252 PMC4587544

[37] Khemlina G, Ikeda S, Kurzrock R. The biology of hepatocellular carcinoma: implications for genomic and immune therapies. Mol Cancer 2017;16:149. 28854942 10.1186/s12943-017-0712-x PMC5577674

[38] Capurro M, Martin T, Shi W, Filmus J. Glypican-3 binds to Frizzled and plays a direct role in the stimulation of canonical Wnt signaling. J Cell Sci 2014;127:1565–75. 24496449 10.1242/jcs.140871

[39] Li N, Wei L, Liu X, A frizzled-like cysteine-rich domain in glypican-3 mediates wnt binding and regulates hepatocellular carcinoma tumor growth in mice. Hepatology 2019;70:1231–45. 30963603 10.1002/hep.30646 PMC6783318

[40] Wei Y, Van Nhieu JT, Prigent S, Srivatanakul P, Tiollais P, Buendia MA. Altered expression of E-cadherin in hepatocellular carcinoma: correlations with genetic alterations, beta-catenin expression, and clinical features. Hepatology 2002;36:692–701. 12198663 10.1053/jhep.2002.35342

[41] Finch PW, He X, Kelley MJ, Purification and molecular cloning of a secreted, Frizzled-related antagonist of Wnt action. Proc Natl Acad Sci U S A 1997;94:6770–5. 9192640 10.1073/pnas.94.13.6770 PMC21233

[42] Taniguchi K, Roberts LR, Aderca IN, Mutational spectrum of beta-catenin, AXIN1, and AXIN2 in hepatocellular carcinomas and hepatoblastomas. Oncogene 2002;21:4863–71. 12101426 10.1038/sj.onc.1205591

[43] Ding Y, Shen S, Lino AC, Curotto de Lafaille MA, Lafaille JJ. Beta-catenin stabilization extends regulatory T cell survival and induces anergy in nonregulatory T cells. Nat Med 2008;14:162–9. 18246080 10.1038/nm1707

[44] Saegusa M, Hashimura M, Yoshida T, Okayasu I. beta- Catenin mutations and aberrant nuclear expression during endometrial tumorigenesis. Br J Cancer 2001;84:209–17. 11161379 10.1054/bjoc.2000.1581 PMC2363713

[45] Galluzzi L, Spranger S, Fuchs E, López-Soto A. WNT signaling in cancer immunosurveillance. Trends Cell Biol 2019;29:44–65. 30220580 10.1016/j.tcb.2018.08.005 PMC7001864

[46] Fujita M, Yamaguchi R, Hasegawa T, Classification of primary liver cancer with immunosuppression mechanisms and correlation with genomic alterations. EBioMedicine 2020;53:102659. 32113157 10.1016/j.ebiom.2020.102659 PMC7048625

[47] Guichard C, Amaddeo G, Imbeaud S, Integrated analysis of somatic mutations and focal copy-number changes identifies key genes and pathways in hepatocellular carcinoma. Nat Genet 2012;44:694–8. 22561517 10.1038/ng.2256 PMC3819251

[48] Fan X, Jin S, Li Y, Genetic and epigenetic regulation of E-cadherin signaling in human hepatocellular carcinoma. Cancer Manag Res 2019;11:8947–63. 31802937 10.2147/CMAR.S225606 PMC6801489

[49] Li L, Rao X, Wen Z, Implications of driver genes associated with a high tumor mutation burden identified using next-generation sequencing on immunotherapy in hepatocellular carcinoma. Oncol Lett 2020;19:2739–48. 32218826 10.3892/ol.2020.11372 PMC7068659

[50] Lehwald N, Tao GZ, Jang KY, β-catenin regulates hepatic mitochondrial function and energy balance in mice. Gastroenterology 2012;143:754–64. 22684045 10.1053/j.gastro.2012.05.048 PMC12045480

[51] Kolthammer JA, Corn DJ, Tenley N, PET imaging of hepatocellular carcinoma with 18F-fluoroethylcholine and 11C-choline. Eur J Nucl Med Mol Imaging 2011;38:1248–56. 21344223 10.1007/s00259-011-1743-y

[52] Kwee SA, Sato MM, Kuang Y, [18F]Fluorocholine PET/CT imaging of liver cancer: radiopathologic correlation with tissue phospholipid profiling. Mol Imaging Biol 2017;19:446–55. 27787742 10.1007/s11307-016-1020-3 PMC5407951

[53] Colnot S, Decaens T, Niwa-Kawakita M, Liver-targeted disruption of Apc in mice activates beta-catenin signaling and leads to hepatocellular carcinomas. Proc Natl Acad Sci U S A 2004;101:17216–21. 15563600 10.1073/pnas.0404761101 PMC535370

[54] Kwee SA, Tiirikainen M, Sato MM, Transcriptomics associates molecular features with ^18^F-fluorocholine PET/CT imaging phenotype and its potential relationship to survival in hepatocellular carcinoma. Cancer Res 2019;79:1696–704. 30760520 10.1158/0008-5472.CAN-18-3837 PMC6494445

[55] Jaiswal S, Ebert BL. Clonal hematopoiesis in human aging and disease. Science 2019;366:eaan4673. 31672865 10.1126/science.aan4673 PMC8050831

[56] Razavi P, Li BT, Brown DN, High-intensity sequencing reveals the sources of plasma circulating cell-free DNA variants. Nat Med 2019;25:1928–37. 31768066 10.1038/s41591-019-0652-7 PMC7061455

[57] Buscarlet M, Provost S, Zada YF, *DNMT3A* and *TET2* dominate clonal hematopoiesis and demonstrate benign phenotypes and different genetic predispositions. Blood 2017;130:753–62. 28655780 10.1182/blood-2017-04-777029

[58] Loberg MA, Bell RK, Goodwin LO, Sequentially inducible mouse models reveal that Npm1 mutation causes malignant transformation of Dnmt3a-mutant clonal hematopoiesis. Leukemia 2019;33:1635–49. 30692594 10.1038/s41375-018-0368-6 PMC6609470

[59] Patra KC, Kato Y, Mizukami Y, Mutant GNAS drives pancreatic tumourigenesis by inducing PKA-mediated SIK suppression and reprogramming lipid metabolism. Nat Cell Biol 2018;20:811–22. 29941929 10.1038/s41556-018-0122-3 PMC6044476

[60] Nomura R, Saito T, Mitomi H, GNAS mutation as an alternative mechanism of activation of the Wnt/β-catenin signaling pathway in gastric adenocarcinoma of the fundic gland type. Hum Pathol 2014;45:2488–96. 25288233 10.1016/j.humpath.2014.08.016

[61] Romero D, Iglesias M, Vary CP, Quintanilla M. Functional blockade of Smad4 leads to a decrease in beta-catenin levels and signaling activity in human pancreatic carcinoma cells. Carcinogenesis 2008;29:1070–6. 18310088 10.1093/carcin/bgn054

[62] Hoshida Y, Nijman SM, Kobayashi M, Integrative transcriptome analysis reveals common molecular subclasses of human hepatocellular carcinoma. Cancer Res 2009;69:7385–92. 19723656 10.1158/0008-5472.CAN-09-1089 PMC3549578

[63] Boyault S, Rickman DS, de Reyniès A, Transcriptome classification of HCC is related to gene alterations and to new therapeutic targets. Hepatology 2007;45:42–52. 17187432 10.1002/hep.21467

[64] Chiang DY, Villanueva A, Hoshida Y, Focal gains of VEGFA and molecular classification of hepatocellular carcinoma. Cancer Res 2008;68:6779–88. 18701503 10.1158/0008-5472.CAN-08-0742 PMC2587454

[65] Huang X, Yang C, Wang J, Sun T, Xiong H. Integrative analysis of DNA methylation and gene expression reveals distinct hepatocellular carcinoma subtypes with therapeutic implications. Aging (Albany NY) 2020;12:4970–95. 32201399 10.18632/aging.102923 PMC7138576

[66] Li T, Fan J, Wang B, TIMER: a web server for comprehensive analysis of tumor-infiltrating immune cells. Cancer Res 2017;77:e108–10. 29092952 10.1158/0008-5472.CAN-17-0307 PMC6042652

[67] Tan PS, Nakagawa S, Goossens N, Clinicopathological indices to predict hepatocellular carcinoma molecular classification. Liver Int 2016;36:108–18. 26058462 10.1111/liv.12889 PMC4674393

[68] Qiu J, Xu J, Zhang K, Refining cancer management using integrated liquid biopsy. Theranostics 2020;10:2374–84. 32089746 10.7150/thno.40677 PMC7019147

[69] Huang da W, Sherman BT, Lempicki RA. Systematic and integrative analysis of large gene lists using DAVID bioinformatics resources. Nat Protoc 2009;4:44–57. 19131956 10.1038/nprot.2008.211

[70] Ramapriyan R, Caetano MS, Barsoumian HB, Altered cancer metabolism in mechanisms of immunotherapy resistance. Pharmacol Ther 2019;195:162–71. 30439456 10.1016/j.pharmthera.2018.11.004

[71] Wei R, Wang J, Wang X, Clinical prediction of HBV and HCV related hepatic fibrosis using machine learning. EBioMedicine 2018;35:124–32. 30100397 10.1016/j.ebiom.2018.07.041 PMC6154783

[72] Wang T, Yang X, Tang H, Integrated nomograms to predict overall survival and recurrence-free survival in patients with combined hepatocellular cholangiocarcinoma (cHCC) after liver resection. Aging (Albany NY) 2020;12:15334–58. 32788423 10.18632/aging.103577 PMC7467372

[73] Chen W, Samuelson FW, Gallas BD, Kang L, Sahiner B, Petrick N. On the assessment of the added value of new predictive biomarkers. BMC Med Res Methodol 2013;13:98. 23895587 10.1186/1471-2288-13-98 PMC3733611

[74] Cook NR. Quantifying the added value of new biomarkers: how and how not. Diagn Progn Res 2018;2:14. 31093563 10.1186/s41512-018-0037-2 PMC6460632

[75] Benson AB, D’Angelica MI, Abbott DE, Guidelines insights: hepatobiliary cancers, Version 2.2019. J Natl Compr Canc Netw 2019;17:302–10. 30959462 10.6004/jnccn.2019.0019

